# Angiotensin Converting Enzyme 1 Expression in the Leukocytes of Adults Aged 64 to 67 Years

**DOI:** 10.2196/45220

**Published:** 2023-01-20

**Authors:** Valquiria Bueno, Pedro Henrique Destro, Daniela Teixeira, Daniela Frasca

**Affiliations:** 1 Division of Immunology Department of Microbiology Immunology and Parasitology Federal University of São Paulo São Paulo Brazil; 2 Department of Microbiology and Immunology Miller School of Medicine University of Miami Miami, FL United States

**Keywords:** aging, angiotensin converting enzyme, lymphocytes, immunosenescence, inflammaging

## Abstract

The renin angiotensin system is composed of several enzymes and substrates on which angiotensin converting enzyme (ACE) 1 and renin act to produce angiotensin II. ACE1 and its substrates control blood pressure, affect cardiovascular and renal function, hematopoiesis, reproduction, and immunity. The increased expression of ACE1 has been observed in human monocytes during congestive heart failure and abdominal aortic aneurysm. Moreover, T lymphocytes from individuals with hypertension presented increased expression of ACE1 after in vitro stimulation with angiotensin II (ATII) with the highest ACE1 expression observed in individuals with hypertension with low-grade inflammation. Our group and others have shown that aging is associated with comorbidities, chronic inflammation, and immunosenescence, but there is a lack of data about ACE1 expression on immune cells during the aging process. Therefore, our aim was to evaluate the levels of ACE1 expression in nonlymphoid cells compared to lymphoid that in cells in association with the immunosenescence profile in adults older than 60 years. Cryopreserved peripheral blood mononuclear cells obtained from blood samples were used. Cells were stained with monoclonal antibodies and evaluated via flow cytometry. We found that ACE1 was expressed in 56.9% of nonlymphocytes and in more than 90% of lymphocytes (all phenotypes). All donors exhibited characteristics of immunosenescence, as evaluated by low frequencies of naïve CD4^+^ and CD8^+^ T cells, high frequencies of effector memory re-expressing CD45RA CD8^+^ T cells, and double-negative memory B cells. These findings, in addition to the increased C-reactive protein levels, are intriguing questions for the study of ACE1, inflammaging, immunosenescence, and perspectives for drug development or repurposing (Reviewed by the Plan P #PeerRef Community).

## Introduction

**Figure 1:**
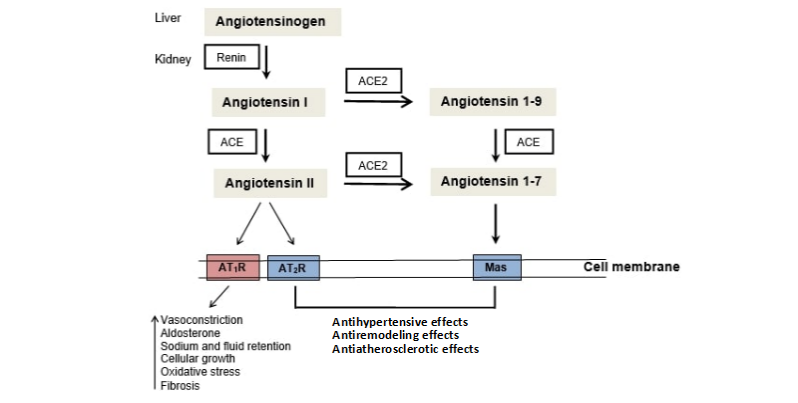
The renin angiotensin system. ACE: angiotensin converting enzyme; ACE1: angiotensin converting enzyme 1; AGTR1: angiotensin II type 1 receptor; AGTR2: angiotensin II type 2 receptor.

Angiotensin converting enzyme (ACE1, also known as CD143) and renin are components of the renin angiotensin system (RAS) acting to produce angiotensin II. In a simplistic definition, RAS is composed of a vasoconstrictor, proinflammatory ACE1/angiotensin II (ATII)/ATII receptor type 1 (AGTR1) axis, and a vasodilating anti-inflammatory ACE2/angiotensin-(1-7) [Ang-(1-7)]/Mas receptor axis (Figure 1). In addition to blood pressure control, ACE1 and its peptide substrates affect cardiovascular and renal function, hematopoiesis, reproduction, and the immunity [1,2]. Thus, it seems crucial that the RAS presents an inflammatory axis and an anti-inflammatory axis for adequate regulation of the immune response. ACE1 expression has been not only observed in tissues, but also its soluble form has been found in urine, serum, seminal fluid, amniotic fluid, and cerebrospinal fluid [3].

The expression of ACE1 in cells from the immune system has been reported in health and disease. Costerousse et al [4] observed, via reverse transcriptase–polymerase chain reaction and southern blot analysis, the expression of ACE1 in monocytes, macrophages, and T cells but not in B cells in healthy adult donors. In addition, ACE1 activity was very low in monocytes, whereas it was high in macrophages (monocytes driven to differentiation). T cells presented intermediary ACE1 activity and B cells expressed no activity [4]. In patients with type 1 diabetes (median age 29 years, normotension), higher *ACE1* and lower *ACE2* expression were observed when compared to healthy controls (median age 32 years, normotension) [5]. Coppo et al [6] found that T cells in culture had increased mRNA expression of *ACE1* and *AGTR1* in individuals with obesity with low-grade inflammation (high-sensitivity C-reactive protein [CRP] level of >3 mg/dL). ACE1 activity was also increased in the supernatant of a T cell culture in individuals with obesity with a high-sensitivity CRP level of >3 mg/dL. Moreover, expression of RAS genes in T cells and levels of inflammatory cytokines in the serum were oppositely associated with serum levels of insulin [6,7]. Ulrich et al [8] have shown that the increased expression of ACE1 in monocytes was associated with kidney and cardiovascular disease progression, suggesting that circulating leukocytes can modulate local immune responses via their own RAS components [8-10].

Considering that aging has been associated with comorbidities, low-grade chronic inflammation, and altered frequency or function of immune cells [11-14], it seems reasonable to suggest that ACE1 play an important role in the aging process. ACE1 has been suggested to influence age-related diseases (ie, Alzheimer disease, sarcopenia, and cancer) but the associated mechanisms are still under investigation. *ACE1* polymorphisms have been correlated with susceptibility to Alzheimer disease [15,16]. In addition, it was shown recently that in normal aging, ACE1 expression is increased in brain homogenates, and this expression is unchanged in early stages of Alzheimer disease [17]. Regarding sarcopenia, Yoshihara et al [18] found a weak correlation between *ACE1* polymorphism and physical function. In cancer (gastric or colorectal), patients presented higher expression of ACE1 in tumors than in healthy tissues [19,20]. In hematopoietic stem/progenitor cells isolated from peripheral blood, Joshi et al [21] showed that aging is associated with decreased ACE2 and increased ACE1 protein expression. This imbalance suggests a bias to the detrimental proinflammatory axis of the local RAS. Considering the scarce information about ACE1 expression in the phenotypes of T and B cells, we aimed to investigate ACE1 expression in cells from the immune system and parameters of immunosenescence in adults older than 60 years. Results herein show different levels of expression of ACE1 in nonlymphoid versus lymphoid cells, with expression being higher in lymphoid cells.

## Methods

### Overview

Blood was collected from adults (n=6, four females and 2 males) aged 64-67 years in 2015. Peripheral blood mononuclear cells were isolated using a Ficoll–Hypaque density gradient (Amersham Biosciences) and centrifugation. Viable cells were counted, adjusted to 2×106/100 μL in 80% fetal bovine serum and 20% dimethylsulfoxide (Sigma), and frozen stored until the phenotyping. In 2021, cells were thawed, checked for viability, and stained with monoclonal antibodies to the T cell phenotypes CD4 PerCP Cy5.5, CD8 APC Cy7, CD27 APC, CD45RA PE; B cell phenotypes CD19 PE, CD27 APC, IgD PE Cy5.5 (eBioscience), and ACE CD143 fluorescein isothiocyanate (R&D Systems). After 30 minutes of incubation with monoclonal antibodies in the dark at 4 °C, the cells were washed with phosphate-buffered saline and centrifuged. Living cells (based on forward and side scatter) were acquired in the BD FACSCanto II flow cytometry system using the DIVA software (Becton Dickinson).

For assessing metabolic parameters, the serum of studied individuals was previously isolated through centrifugation and frozen stored until use. Measurement of metabolic parameters was performed in the Laboratório Central–Hospital São Paulo, Federal University of São Paulo.

### Statistical Analysis

Data are presented as mean (SD) values. To test the normality of data, we used the Shapiro-Wilk test. We considered *P* values for interindividual differences in each variable, since individuals were aged differently (biological aging) and thus, physiological parameters could be affected by genetics, lifestyle, nutrition, and comorbidities. A *P* value less than .05 was considered significant.

### Ethics Approval

The Ethics Committee of the Federal University of São Paulo approved all procedures (protocol 10904).

## Results

Table 1 shows that older adults are heterogeneous for some physiological parameters such as glucose, urea, glycated hemoglobin, and CRP.

**Figure 2:**
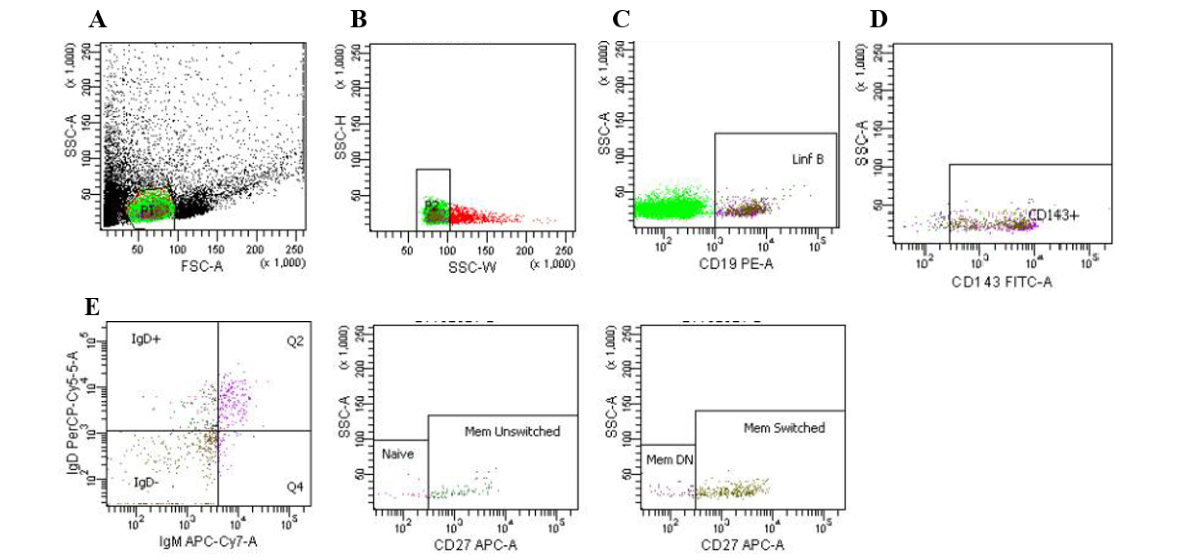
Flow cytometry gating strategy for B cell phenotypes and CD143 expression. (A) All cells and gates for lymphocyte (green) based on forward scatter (FSC-A) and side scatter (SSC-A); (B) exclusion of doublets (from the lymphocyte gate); (C) CD19^+^ B cells (from the doublets exclusion gate); (D) CD143+ACE1 cells (from the CD19^+^ B cells’ gate); and (E) B cell phenotypes and CD143+-IgM+IgD+CD27- (naïve), IgMlowIgD-CD27^+^ (memory-unswitched), IgM-IgD-CD27^+^ (memory-switched), and IgM+IgD-CD27- (memory double-negative). DN: double-negative; FSC: forward scatter; Mem: memory; SSC: side scatter.

**Figure 3:**
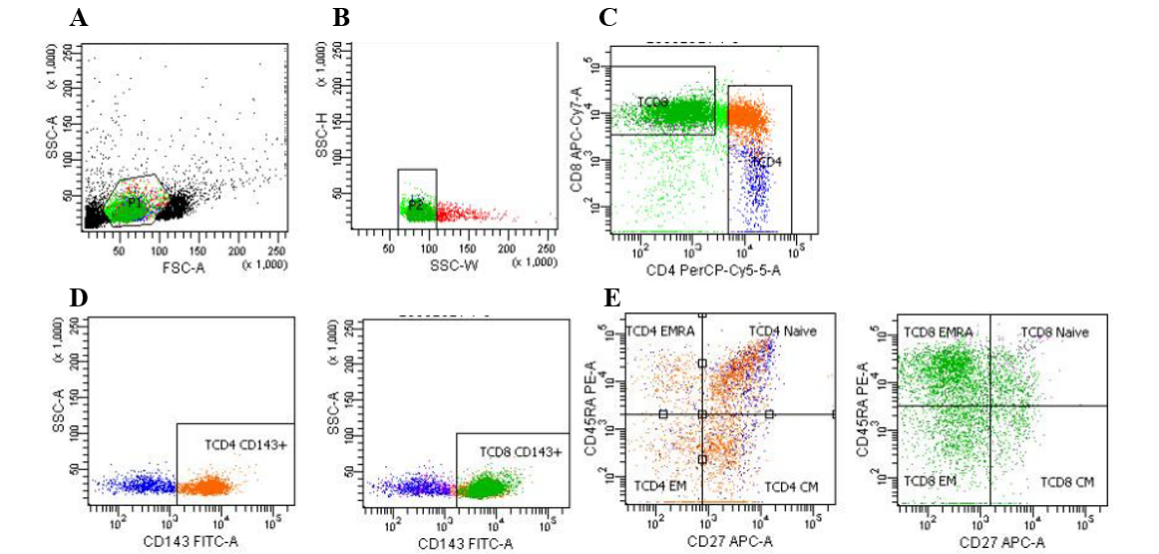
Flow cytometry gating strategy for T cell phenotypes and CD143 expression. (A) All cells and gates for lymphocyte (green) based on forward scatter (FSC-A) and side scatter (SSC-A); (B) exclusion of doublets (from the lymphocyte gate); (C) CD4^+^ and CD8^+^ T cells (from the doublets exclusion gate); (D) CD143+ACE1 cells (from the CD4^+^ and CD8^+^ T cells’ gate); (E) T cell phenotypes and CD143^+^, CD45RA+CD27- (naïve), CD45RA-CD27^+^ (central memory), CD45RA-CD27- (effector memory), and CD45RA+CD27- (effector memory re-expressing CD45RA) cells. FSC: forward scatter; SSC: side scatter.

**Figure 4:**
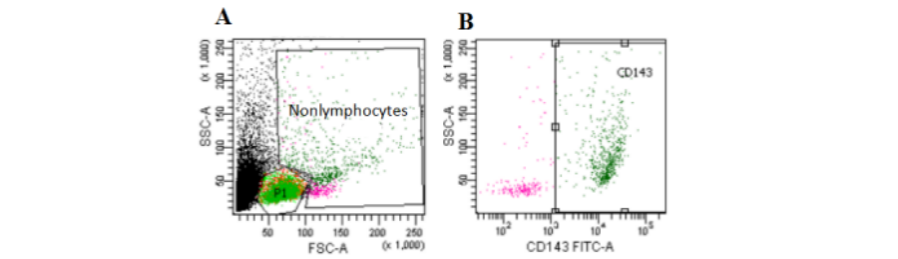
Flow cytometry gating strategy for nonlymphocytes and CD143 expression. (A) All cells and gates for lymphocytes (P1) and nonlymphocytes based on forward scatter (FSC-A) and side scatter (SSC-A) and (B) CD143^+^ ACE1 cells (from the nonlymphocyte gate). FSC: forward scatter; SSC: side scatter.

Table 2 and Figures 2-4 show that CD143 (ACE1) is expressed in almost 100% of lymphocytes, whereas it is expressed in 56.9% (SD 20.6%) of nonlymphocytes. CD8^+^ T cells presented the highest expression (98.4%), followed by CD19^+^ B cells (93.7%, SD 3.4%) and CD4^+^ T cells (90.7%, SD 8.7%). In T cells, ACE1 is expressed in all phenotypes (naïve, central memory, effector memory, and effector memory re-expressing CD45RA [EMRA]). In B cells, ACE1 was expressed in naïve, unswitched memory, switched memory, and double-negative (DN) cells.

Table 3 shows that characteristics of senescent T cells were observed in both males and females, such as low expression in naïve CD4^+^ and CD8^+^ T cells and high expression in EMRA CD8^+^ T cells.

Table 4 shows that aging adults with lower percentages of naïve B cells also presented a higher percentage of DN memory B cells.

**Table 1:** Physiological parameters observed in older adults.

	Cholesterol^a^ (mg/dL)	Low-density lipoprotein^a^ (mg/dL)	Triglycerides^a^ (mg/dL)	Glucose^b^ (mg/dL)	Urea^c^ (mg/dL)	Creatinine^a^ (mg/dL)	Albumin^a^ (mg/dL)	Glycated hemoglobin^d^ (mg/dL)	C-reactive protein^e^ (mg/dL)
Individual participants’ values	207, 253, 181, 223, 249, and 191	137, 176, 96, 150, 186, and 125	152, 152, 130, 149, 163, and 130	80, 86, 137, 83, 89, and 165	30, 40, 28, 28, 29, and 28	0.86, 0.73, 0.84, 0.68, 0.79, and 1.01	3.8, 4.1, 3.2, 4.2, 3.8, and 3.4	5.9, 6.2, 7.9, 5.5, 5.8, and 6.0	7.3, 4.1, 6.0, 23.1, 4.6, and 0.6
Overall, mean (SD)	217.3 (27.2)	145.0 (30.4)	146.0 (12.1)	106.7 (32.5)	30.5 (4.3)	0.82 (0.1)	3.8 (0.4)	6.2 (0.8)	7.6 (7.2)

^a^*P*>.10.

^b^*P*=.047.

^c^*P*=.02.

^d^*P*=.02.

^d^*P*=.03.

**Table 2:** CD143 (ACE1) expression in lymphocytes and nonlymphocytes.

	Lymphocytes (%)				Nonlymphocytes^a^ (%)
	CD4^+^CD143^+b^	CD8^+^CD143^+b^	CD19^+^CD143^+^^b^		
Individual participants’ values	84.8, 77.6, 96.9, 98.8, 87.8, and 98.3	97.1, 96.7, 99.0, 99.6, 98.5, and 99.6	90.5, 90.6, 91.4, 99.0, 95.7, and 94.9	74.6, 35.4, 47.7, 75.0, 32.9, and 75.9	
Overall, mean (SD)	90.7 (8.7)	98.4 (1.3)	93.7 (3.4)	56.9 (20.6)	

^a^*P*=.08.

^b^*P*>.15.

**Table 3:** Phenotypes of CD4^+^ and CD8^+^ T cells.

	CD4^+^ T cells (%)					CD8^+^ T cells (%)			
	Naïve^a^	Central memory^b^	Effector memory^a^	Effector memory re-expressing CD45RA^b^	Naïve^a^		Central memory^a^	Effector memory^a^	Effector memory re-expressing CD45RA^a^
Individual participants’ values	27.6, 43.3, 13.4, 12.5, 24.8, and 32.6	55.9, 29.1, 55.4, 49.8, 55.3, and 25.4	12.4, 15.4, 29.2, 34.7, 18.3, and 19.7	4.1, 12.2, 2.0, 3.0, 1.5, and 22.4	17.3, 10.2, 13.6, 10.7, 12.8, and 11.7		26.5, 6.5, 10.3, 16.6, 11.5, and 18.3	20.1, 24.8, 13.6, 9.8, 27.6, and 20.4	36.0, 58.6, 62.5, 63.0, 48.1, and 49.6
Overall, mean (SD)	25.7 (11.7)	45.2 (14.1)	21.6 (8.6)	7.5 (8.3)	12.7 (2.6)		15.0 (7.1)	19.4 (6.7)	53.0 (10.4)

^a^*P*>.10.

^b^*P*=.047.

**Table 4:** Phenotypes of CD19^+^ cells.

	Naïve^a^ (%)	Unswitched memory^a^ (%)	Switched memory^a^ (%)	Double-negative memory^a^ %
	73.8, 61.3, 28.6, 51.8, 35.9, and 67.7	6.3, 6.9, 4.1, 10.0, 7.9, and 3.5	4.0, 5.7, 31.4, 22.1, 18.5, and 9.8	15.9, 26.1, 35.8, 16.1, 37.7, and 19.0
Overall, mean (SD)	53.2 (17.9)	6.5 (2.4)	15.3 (10.6)	25.1 (9.8)

^a^*P*>.10.

## Discussion

Our results show that for the studied population, chronological aging and biological aging are asynchronous. Even among individuals with a small chronological difference (64 to 67 years), there is heterogeneity in physiological parameters such as glucose, urea, glycated hemoglobin, and CRP. Changes in the same functional parameters have been reported by Carlsson et al [22] and Helmerson-Karlqvist [23] in healthy older adults. Carlsson’s [22] study found that the CRP level was 2.6% with a coefficient variation of 1.4%, whereas in our study, we observed higher levels of CRP in 5 out of 6 individuals. Increased CRP levels have been associated with inflammaging, and our findings show that the study population has changes in functional parameters, which are likely associated with an inflammatory profile [24].

The link between the RAS and inflammation has been suggested but its role is not completely clear under physiological and pathological conditions [25,26]. In addition, the association between altered ACE1 expression in tissues (brain, muscle, heart, and vessels) and the development and progression of age-related conditions such as Alzheimer disease, sarcopenia, and cardiovascular disease has been suggested, but results are controversial [17,27-30].

There are few studies showing the association between ACE1 expression in cells from the immune system (monocytes and T cells) and the progression of kidney and cardiovascular disease [8,9,31,32]. Therefore, considering the lack of information on this issue, we questioned whether ACE1 (CD143) was highly expressed in cells from the immune system during the aging process. We found that ACE1 was expressed in almost 100% of T (CD4^+^ and CD8^+^) and B lymphocytes and in all phenotypes of these cells. In nonlymphoid cells, mean ACE1 expression was 56.9% (SD 20.6%). In agreement with our findings, independent studies showed that T cells from healthy donors and monocytes from patients with congestive heart failure expressed ACE1, but there has been no investigation on cell phenotypes [25,26]. Our study is the first to show that either inexperienced (naïve) or fully activated (memory) cells expresses ACE1. Our findings suggest that the expression of ACE1 in lymphoid and nonlymphoid cells reflects health status, since our studied population presented changes in physiological parameters and high levels of ACE1 expression in immune cells. Previous independent studies showed that patients with unstable angina [32] or acute myocardial infarction [33] presented higher expression of ACE1 in T cells and dendritic cells than control subjects. In addition, markers of cell (lymphoid and nonlymphoid) functional status, such as inflammatory or growth factor production, could be modulated by ACE inhibitors (ACEi). Accordingly, mononuclear leukocytes from healthy subjects incubated with an endotoxin exhibited high levels of tissue factor activity, which was reduced in the presence of captopril in a dose-dependent pattern. This result could be related to the antithrombotic effect of ACEi [34]. In patients with congestive heart failure, immune cells cultured with lipopolysaccharide secreted high levels of the proinflammatory tumor necrosis factor α, and these levels were significantly reduced in the presence of captopril [35].

It may be proposed that mechanistically, ATII is produced by mononuclear cells or lymphocytes and, at the same time, ATII induces immunologic activation in these cells. Therefore, the inflammatory axis ACE1/ATII/AGTR1 and the counterregulator ACE2/Ang-(1-7)/Mas receptor axis [36,37] could play a role in chronic diseases, inflammaging, and immunosenescence observed in older adults. Our studied population presented changes in some physiological parameters and increased levels of CRP. This inflammatory profile [24], in addition to more than 90% of T and B cells expressing ACE1 in our population of older adults, suggest a correlation among aging, inflammaging, and ACE1 expression. Independent of chronological age, inflammation (even if related to subclinical diseases) may be a contributor to disease progression when the balance with anti-inflammation is shifted [38]. In this context, the regulation of ACE1/ACE2 expression could be explored as a target for the balance of exacerbated inflammatory reactions. Considering that the equilibrium between ACE1 and ACE2 expression could play an important role in healthy aging, our subsequent studies will be focused on ACE1 and ACE2 expression in cells from the immune system.

The phenotype of T and B lymphocytes has been used to identify senescence in immune cells. CD4^+^ T cells present changes during the aging process with a decrease in naïve phenotypes and an increase in effector memory phenotypes, whereas CD8^+^ T cells show a decrease in the naïve phenotype and an increase in the effector memory and EMRA phenotypes [12,39,40]. It has been shown that the reduction in naïve B cells is accompanied by no change in memory-unswitched and memory-switched B cells but an increase in the percentage of double-negative B cells [41-44]. Using these phenotypes, we found a similar senescent phenotype in some of the studied aging adults. The reduction in naïve lymphocytes has been related to impaired antigen responsiveness, and for B cells, a decrease in the production of antibodies has been observed [45,46]. The increased percentage of DN memory B cells has been linked to autoimmune diseases [47,48]. We observed ACE1 expression in more than 90% of T cells and B cells and in all phenotypes. ACE1 was expressed in nonlymphocytes in a range of 32.9% to 75.9%. Our findings suggest that ACE1 could play a role in several processes linked to aging, including the generation and activation of autoimmune cells, due to the experimental evidence that inhibitors of ACE1 suppress the autoimmune process in a number of autoimmune diseases such as experimental autoimmune encephalomyelitis, arthritis, autoimmune myocarditis [49].

This study is the first to compare the expression of the protein ACE1 between different cell types, both lymphoid cells (CD4^+^ and CD8^+^ T cells and B cells) and nonlymphocytes in older adults. It was also observed that even though the study participants were in the early stage of chronological aging (64 to 67 years), they presented heterogeneity in physiological parameters, signs of inflammaging (increased CRP levels), and immunosenescence, including low expression in naïve T and B cells in addition to the accumulation of terminally differentiated CD8^+^ T cells and DN B cells. This study has limitations such as the small sample size and the lack of young adults for comparison. As an example, the subject presenting the highest CRP and albumin levels also exhibited a high percentage of ACE1 expression in T cells (CD4^+^ and CD8^+^), B cells, and nonlymphoid cells, in addition to the lowest percentage of CD4^+^ naïve cells, and the highest percentage of CD8^+^ terminally differentiated (EMRA) and DN B cells. However, due to the small sample size, it was not possible to associate the high expression of ACE1 in immune cells with inflammaging and immunosenescence. Correlation of physiological parameters and health status with ACE1 expression and investigating whether age and associated chronic diseases could lead to increased ACE1 expression would yield important information. Moreover, we only determined CRP as a marker of inflammaging, and interleukin 6 and tumor necrosis factor α would be desirable to complete our panel. Functional analyses are needed to clarify the impact of ACE1 expression on immune cells and whether ACEi and angiotensin receptor blockers administered to patients with hypertension somehow affect immunity. Recently, it was shown that membrane-bound ACE2 acts as a receptor for SARS-CoV-2, but the possible effects on RAS components [ATII, Ang-(1-7), ACE1, ACE2, AT1, and Mas] and whether ACEi and angiotensin receptor blockers interfere with the mitigation of COVID-19 require further investigation [50-54]. Therefore, it is important to emphasize the negative impact of chronic diseases on the outcomes of older adults during a viral infection and how ACE1 or ACE2 expression in immune cells could provide information regarding diagnosis and treatment.

## Abbreviations

ACE1: angiotensin converting enzyme
ACEi: angiotensin converting enzyme inhibitors
AGTR1: angiotensin II receptor type 1
Ang-(1-7): angiotensin-(1-7)
ATII: angiotensin II
DN: double-negative
EMRA: effector memory re-expressing CD45RA
RAS: renin angiotensin system
